# Emotion regulation, hope, and optimism during the third wave of the COVID-19 pandemic: The role of age and personality

**DOI:** 10.1371/journal.pone.0296205

**Published:** 2024-01-02

**Authors:** Elena Carbone, Graziana Lenti, Enrico Sella, Angelica Moè, Erika Borella

**Affiliations:** Department of General Psychology, University of Padova, Padova, Italy; University of Macerata: Universita degli Studi di Macerata, ITALY

## Abstract

**Aims:**

This study examined age-related differences between young and older adults’ emotion regulation, hope, and optimism 1 year after the COVID-19 outbreak. Whether personality explained such outcomes was also examined.

**Method:**

A sample of 228 young adults and 161 older adults was interviewed in April-May 2021 to complete questionnaires assessing cognitive reappraisal (CR) and expressive suppression (ES) emotion regulation strategies use, optimism, hope (agency and pathways components), and personality traits.

**Results:**

Older adults reported greater CR and ES use, optimism, and hope-agency levels than young adults, whereas no age differences emerged for hope-pathway scores. Personality traits (more consistently emotional stability) contributed to explaining CR and ES use, and greater hopeful and optimistic dispositions.

**Conclusions:**

These findings confirm older adults’ advantage in facing the emotional and psychological fallout of the COVID-19 pandemic in its third wave. They also underscore the importance of considering personality to depict individual profiles prone to experiencing long-term negative emotional/psychological consequences of emergencies as COVID-19.

## Introduction

COVID-19 infection disproportionately affected older adults, compared to younger people, in the first acute phase of the pandemic (from March to June 2020). Despite the age-related vulnerability to COVID-19 infection, under the first lockdown older adults reported better psychological well-being, lower levels of distress and negative affect, higher social connectedness, and lower levels of loneliness than young adults did [1–3]. Such an older adults’ advantage in managing the highly complex situation could have been theoretically predicted, standing from aging theories developed to explain age-related changes in emotional functioning in normal times. The above findings are in fact in line with the assumptions of socioemotional selectivity theory (SST) [4]. According to SST, despite age-related losses in different domains (e.g., physical and cognitive), the tendency to prioritize positive emotional meanings, goals, and experiences increases with aging. Motivational shifts and perceived time constraints accordingly contribute to explaining older adults’ overall better regulation of emotions or psychological outcomes compared to young adults [5], which improves their emotional and psychological well-being also during very stressful events [1–3]. It has been argued that more favorable emotional and psychological responses with aging, even when facing negative experiences, might lie in age-related changes in older adults’ use of emotion regulation strategies [5, 6]. Among the common forms of emotion regulation, cognitive reappraisal (CR) involves thinking of ways to alter a situation’s meaning and emotional effect, and expressive suppression (ES) is the attempt to inhibit or reduce ongoing emotion-expressive behavior. CR can be adaptive, whereas ES is argued to be maladaptive. In fact, CR typically relates to higher levels of positive affect and greater psychological well-being, and ES is expected to relate to increased negative affect or psychopathological symptoms (e.g., depression and anxiety) [7]. Compared to their younger counterparts, older adults have been found more likely to employ the adaptive CR strategy. Mixed results emerge from the ES strategy. Studies have found that older adults are less likely to employ ES than young adults [5, 6]. Other studies have reported a greater use of ES in older adults compared to young adults. However, the use of ES has not been found to increase adverse well-being outcomes (e.g., psychological distress) in older people relative to younger people [8, 9].

Only one study compared younger and older adults’ use of emotion regulation strategies during an exceptional emergency time as the first wave of the COVID-19 pandemic (between March and June 2020) [10]. The results showed that older adults made greater use of adaptive emotion regulation strategies such as CR than younger adults did to upregulate positive emotions, accept the negative situation at hand, and focus on its positive aspects. No age differences emerged for ES [10]. Although the results emerged from only one study, they align with the evidence of older people’s more flexible and effective use of emotion regulation strategies in the face of the unexpected, stressful context of the COVID-19 pandemic. However, little is known about age-related differences between young and older adults’ emotion regulation with respect to the long-term effects of the pandemic.

Alongside the emotional effects of the COVID-19 pandemic and its related restrictions, concerns related to the pandemic are likely to have affected other psychological outcomes that have a key role in individuals’ psychological well-being and mental health, such as optimism and hope [11]. Dispositional optimism (i.e., an individual’s general tendency to believe that good things will happen in the future) [12, 13] usually increases incrementally from young adulthood to midlife and subsequently declines in late adulthood [14, 15]. Hope is related more to goal-directed behavior and defined as “a cognitive set that is based on a reciprocally derived sense of successful agency (goal-directed determination) and pathways (planning of ways to meet goals)” [16]. Younger and middle-aged people usually experience greater hope than older people do [17], and when agency and pathway components are examined, young adults report more pathways compared to adults (aged 55 to 64 years), whereas no age differences emerge in agency [18].

The few studies that examined age differences between young and older adults in these aspects during the first phases of the COVID-19 pandemic found that older adults were the ones who reported more positive attitudes towards the future than younger adults did [19, 20]. Specifically, in comparison to their younger counterparts, older adults were found to display greater optimism in terms of generalized positive expectations of personal future outcomes and potential resolution of the lived emergency. They also reported a lower sense of hopelessness about the future (although this dimension was examined with only a single-item question), believing that it would be possible to reach personal goals and that the situation could change for the better. These results highlight an older adults’ advantage in coping with the emergency in less threatening ways compared to their younger counterpart [20], at least during the initial phases of the pandemic.

Moreover, individual differences in emotional and psychological functioning, even in stressful situations [21], could be linked, among other factors, also to personality. Evidence shows that personality traits (as framed by the Big Five model [22]) and emotion regulation strategies are interrelated. Lower levels of neuroticism (i.e., higher emotional stability) usually show the largest association with greater use of typically adaptive emotion regulation strategies and lower use of typically less adaptive ones, with all other traits following the same pattern of associations, though with small to moderate effects [23]. Higher levels of emotional stability, extraversion, agreeableness, and conscientiousness were shown to be positively associated with dispositional optimism [24]. Evidence also shows neuroticism (i.e., lower emotional stability) negatively associated with hope, whereas conscientiousness and extraversion positively associated with hope [25, 26].

The few studies conducted across the adult life span assessing personality–emotional and psychological functioning associations during the first phases of the COVID-19 pandemic [2, 27–29] found high emotional stability and extraversion associated with greater use of adaptive emotion regulation strategies and lower use of less adaptive strategies [29]. High emotional stability, extraversion, and conscientiousness were also found to be related to optimistic estimates (i.e., shorter duration) of how long the pandemic would last [29].

Young and older adults’ emotional and psychological functioning seems to have received little attention in the context of a worldwide emergency, such as the COVID-19 pandemic. It is worth mentioning that contagion waves lasted throughout 2020 and 2021, protracting the emergency and resulting in mandatory periods of home confinement and social distancing. Nonetheless, few studies have been conducted to examine the emotional and psychological responses to such a prolonged crisis in its various later stages from an age- and individual-differences perspective (e.g., up to October 2020 in Maggi et al. [30]; December 2020 in Carbone et al. [1]; February 2021 in Kluwe-Schiavon et al. [31]; July 2021 in Fields et al. [32]). Although the COVID-19 pandemic has sadly created an unavoidable, prolonged global crisis, it also represents a suitable context to examine further the age-related and individual differences between young and older adults in those emotional and psychological processes likely to influence and differentiate individuals’ psychological responses to adversities, namely emotion regulation, optimism, and hope.

The present study was therefore intended to show how young and older individuals cope with and overcome extreme events with large-scale, prolonged, stressful features, such as the COVID-19 pandemic. To do so, we examined age-related differences between young and older adults’ emotion regulation, optimism, and hope 1 year (April–May 2021) after the declaration of the COVID-19 pandemic in Italy (March 2020), a period that was characterized by the third contagion wave and hence a return to strict social restrictions. We also newly assessed the role of the Big-Five personality traits in explaining individuals’ emotional and psychological functioning in the prolonged context of the COVID-19 pandemic.

For emotion regulation, we focused on CR and ES, which are two common and well-studied emotion regulation strategies [6]. In line with SST [2, 4] and previous evidence under the COVID-19 pandemic [10], we expected older adults would rely on greater use of the more adaptive CR strategy than young adults would, and we expected no age-related differences in the use of the supposedly less adaptive ES strategy [10].

In light of the findings from studies during the first phases of the COVID-19 pandemic [19, 20], we also hypothesized that older adults would show greater optimistic attitudes toward the future than their younger counterparts would. As for hope, in line with Toussaint et al. [20], an attenuation of age differences between younger and older adults in favor of the latter was expected. Considering its pathways and agency components, we explored for the first time in the context of the COVID-19 pandemic whether the pathways and agency components of hope might display a pattern of age-related differences between younger and older adults.

Finally, in line with evidence found under the COVID-19 pandemic [27, 29], we expected personality traits, and particularly emotional stability and extraversion, would be associated with emotion regulation strategies [29]. Personality traits, in particular emotional stability, extraversion, and conscientiousness should also be associated with optimism [27]. We explored whether personality traits also relate to hopeful attitudes towards the future. Given the role of gender differences and other socio-demographic characteristics as education in influencing our outcomes of interest [17, 33–36], these aspects were also explored.

## Method

### Participants

Two hundred and twenty-eight young adults (age range: 18–35; 124 females) and 161 older adults (age range: 60–91; 89 females) volunteered for the study. All of the participants were native Italian speakers recruited by word of mouth (i.e., activated local community networks). The inclusion criteria were (a) good physical and mental health, assessed with a semi-structured interview [37] asking participants to report, for example, whether they had a history of psychiatric or neurological disorders or other diseases causing cognitive impairments or visual, auditory, and/ or motor or other physical impairments; (b) absence of depressive symptoms, indicated by scores ≤ 14 on the Beck Depression Inventory–II [38], a self-report including 21 items for the assessment of cognitive, affective, somatic, and vegetative symptoms of depression in young adults, and scores ≤ 5 on the 15-item Geriatric Depression Scale [39], a self-report measure of 15 items developed to assess depressive symptoms in older adults; and (c) for older adults, absence of signs of cognitive impairment, indicated by scores of 9 and above on the short version of the Italian Checklist for the Multidimensional Assessment of the Elderly (ICMAE) [40], a cognitive-functioning screening measure that includes 10 questions assessing temporal and spatial orientation, memory, and executive functions.

Table 1 shows the descriptive statistics of the demographic characteristics and the screening measures by age group. All the older adult participants scored 10 out of 10 on the ICMAE. The two age groups did not differ in gender distribution (χ^2^ = .006, *p* = .94), but the older adults had fewer years of education and more COVID-19 worries (see materials for further details) than young adults (see Table 1).

**Table 1:** Descriptive statistics of socio-demographic and COVID-19 worries by group, and results from group differences.

	Young adults (N = 228)		Older adults (N = 161)		Group differences		
	M	SD	M	SD	F_(1,387)_	*p*	*η* ^*2*^_*p*_
Age	23.90	3.57	68.14	7.73			
BDI-II	6.32	3.82	-	-			
GDS	-	-	2.25	1.50			
Education (years)	15.40	2.37	11.48	4.16	138.87	*p* < .001	.26
COVID-19 worries	49.76	22.14	62.74	20.34	34.70	*p* < .001	.08

Notes. BDI-II: Beck Depression Inventory–II; GDS: Geriatric Depression Scale.

The study was approved by the local ethics committee for psychological research (No. 4107; approval date: 19/04/2021) and performed according to the Declaration of Helsinki (World Medical Association, 2013). Participants were informed about the aim of the study and the confidentiality of the data collection, and they gave their consent to participate.

### Materials

#### Emotion regulation

The Emotion Regulation Questionnaire [7] consists of 10 items assessing emotion regulation using two strategies: CR (i.e., attempts to think about ways to alter the meaning and emotional effects of a situation) measured by six items (e.g., “When I want to feel less negative emotion, I change the way I think about the situation”) and ES (i.e., attempts to inhibit or reduce ongoing emotion-expressive behavior) measured by four items (e.g., “I keep my emotions to myself”). Participants were asked to indicate their agreement with each statement on a 7-point Likert scale (1 = *strongly disagree*; 7 = *strongly agree*). The dependent variables were the sum of the scores on each item for CR and ES (Cronbach α .85 for CR and .72 for ES, in the current sample), with higher scores indicating greater use of the two emotion regulation strategies.

#### Optimism

The Life Orientation Test–Revised (LOT-R) [13] consists of 10 items (with four fillers) that assess respondents’ generalized expectations of positive outcomes (three items; e.g., “In uncertain times, I usually expect the best”) versus negative outcomes (three items; e.g., “I hardly ever expect things to go my way”). Participants were asked to indicate their level of agreement with each statement on a 5-point Likert scale from (1 = *strongly disagree*; 5 = *strongly agree*). The dependent variable was the sum of the scores on six items (Cronbach α = .80 in the current sample), with higher scores indicating greater optimism.

#### Hope

The Hope Scale [16] consists of 12 items (with four fillers) that assess an individual’s level of hope through two subscales: agency, or goal-directed energy (four items; e.g., “I energetically pursue my goals”), and pathways, or planning to accomplish goals (four items; e.g., “Even when others get discouraged, I know I can find a way to solve the problem”). Participants answered each statement on a 7-point Likert scale (1 = *definitely false*; 7 = *definitely true*). The dependent variables were the sum of the scores on each item for the two subscales (Cronbach α = .69 for hope-pathways and .72 for hope-agency in the current sample), with higher scores indicating higher hope agency and pathways levels, respectively.

#### Personality traits

The 10-item Big Five Inventory [41] consists of 10 items that assess the five major personality traits: agreeableness (e.g., “I see myself as someone who is generally trusting”), conscientiousness (e.g., “I see myself as someone who does a thorough job”), emotional stability (e.g., “I see myself as someone who is relaxed and handles stress well”), extraversion (e.g., “I see myself as someone who is outgoing and sociable”) and openness (e.g., “I see myself as someone who has an active imagination”). Participants were asked to indicate their agreement with each statement on a 5-point Likert scale (1 = *strongly disagree*; 5 = *strongly agree*). The dependent variables were obtained by averaging the scores on the two items expressing each of the five major personality traits.

#### COVID-related worries

Three ad hoc questions were used to assess COVID-19 worries (“The health emergency over the last year worries you”; “The health emergency over the last year scares you”; and “This third wave scares you more than the previous ones [at the beginning and end of 2020]”). Participants were asked to indicate their agreement with each statement on a scale from 0 (*not at all*) to 100 (*extremely*). The total score was obtained by averaging the scores on each item, with higher scores corresponding to greater COVID-19 worries.

#### Procedure

Participants each took part in a single 90-min phone interview conducted between 20/04/2021 and 20/05/2021. They were asked to participate from a place in a quiet area of their home to avoid hearing issues or distractions. After obtaining the participants’ informed consent, the experimenter guided participants through the completion of the questionnaires, ensuring that they were able to hear and understand the instructions clearly and transcribing their answers, as following: a semi-structured interview assessing demographic characteristics as well as physical and mental health status, the ICMAE (to older adults only), the 10-item Big Five Inventory, the Emotion Regulation Questionnaire, the LOT-R, the Hope Scale, the Beck Depression Inventory–II (for younger adults) or the 15-item Geriatric Depression Scale (for older adults), and the COVID-19 worries questions.

#### Statistical analyses

First, age-related differences between younger and older adults’ CR, ES, hope (agency and pathways), and optimism were examined using a series of analyses of covariance, with age group as the between-subject factor and years of education, gender and COVID-19 worry scores as covariates.

Then, to examine the role of personality in explaining the emotional and psychological outcomes considered here, we ran correlations between all the measures of interest and conducted hierarchical regression analyses. Sociodemographic factors (i.e., age, education, gender) were entered and controlled in Step 1, COVID-19 worries in Step 2, and personality traits in Step 3. CR, ES, hope–agency, hope-pathways, and optimism were entered in different analyses as dependent variables. The analyses were conducted using IBM SPSS (Version 28), and the probability level was set at *p* < .05.

## Results

### Age-related differences

A main effect of age group, in favor of older adults, emerged for CR, ES, optimism, and hope-agency, but not for hope-pathways (see Table 2). Education was a significant covariate for CR and hope-agency. Gender was a significant covariate for ES, whereas COVID-19 worry was a significant covariate for ES and optimism (see Table 2).

**Table 2:** Descriptive statistics of the variables of interest by age group, and results of the ANCOVAs for each measure.

	Young adults (N = 228)	Older adults (N = 161)	ANCOVAs			
	*M (SD)*	*M (SD)*		*F*_*(1*,*384)*_	*p*	*η* ^*2*^_*p*_
Cognitive Reappraisal	28.02 (6.43)	30.03 (6.34)	Age group	12.726	< .001	.032
			*Gender* ^^^	3.785	.052	.010
			*Education*	5.247	.023	.013
			*COVID-19 worries*	<1		
Expressive Suppression	13.59 (4.75)	15.48 (5.04)	Age group	6.613	.010	.017
			*Gender* ^^^	10.069	.002	.026
			*Education*	<1		
			*COVID-19 worries*	6.959	.009	.018
Optimism	13.46 (4.94)	15.38 (3.96)	Age group	19.010	< .001	.047
			*Gender* ^^^	<1		
			*Education*	<1		
			*COVID-19 worries*	5.860	.016	.015
Hope–agency	21.75 (3.10)	22.99 (2.68)	Age group	25.434	< .001	.062
			*Gender* ^^^	<1		
			*Education*	7.975	.005	.020
			*COVID-19 worries*	<1		
Hope–pathways	22.16 (2.99)	22.50 (3.18)	Age group	2.319	.129	.006
			*Gender* ^^^	1.996	.159	.005
			*Education*	<1		
			*COVID-19 worries*	1.393	.239	.004

Notes. Years of education, gender and COVID-19 worries scores were included as covariates.

^^^Gender was a dichotomous variable (0 = female, 1 = male)

### The role of personality

#### Correlations

Table 3 shows the correlations between the assessed variables. Small-to-medium positive correlations emerged between emotional stability, agreeableness, conscientiousness, and openness traits and the CR strategy. A small-to-medium negative correlation emerged between extraversion and the ES strategy, which was also (positively and weakly) correlated with emotional stability and (negatively and weakly) correlated with openness. Positive, small-to-medium correlations emerged between emotional stability, agreeableness, extraversion, and conscientiousness traits and optimism (LOT-R scores). Conscientiousness and emotional stability traits were positively correlated with hope-agency scores, whereas all traits were positively and weakly correlated with hope-pathways scores.

**Table 3:** Matrix of correlations between all the measures of interest.

	1	2	3	4	5	6	7	8	9	10	11	12	13
1. Age	--												
2. Gender^	-.027	--											
3. Education (years)	-.540**	.005	--										
4. COVID-19 worries	.308**	-.197**	-.231**	--									
5. Cognitive Reappraisal	.161**	-.107*	.016	.084	--								
6. Expressive Suppression	.196**	.131**	-.106*	.150**	.012	--							
7. Optimism	.231**	.017	-.071	-.061	.325**	-.194**	--						
8. Hope-agency	.221**	.040	.019	.009	.168**	-.015	.345**	--					
9. Hope-pathways	.046	.085	.004	-.060	.342**	-.087	.365**	.473**	--				
10. Extraversion	.101*	-.032	-.099	.005	.044	-.388**	.262**	.047	.247**	--			
11. Agreeableness	.152**	.019	.012	-.041	.203**	-.077	.279**	.089	.109*	.163**	--		
12. Conscientiousness	.361**	-.067	-.200**	.109*	.116*	.022	.187**	.366**	.176**	.060	.178**	--	
13. Emotional Stability	.089	.230**	.046	-.156**	.208**	.128*	.344**	.154**	.231**	-.001	.150**	.064	--
14. Openness	-.062	.011	.122*	-.072	.120*	-.112*	.067	.059	.184**	.164**	.068	.063	.014

^^^Gender was a dichotomous variable (0 = female, 1 = male)

*p < .05

**p < .01

#### Regression analyses

Tables 4–8 summarize the results from hierarchical regression analyses of each outcome of interest.

**Table 4:** Results from hierarchical regression analysis with age, gender and education (Step 1), COVID-19 worries (Step 2) and personality traits (Step 3) as predictors of cognitive reappraisal.

	Cognitive reappraisal		
	Model 1	Model 2	Model 3
	β	β	β
Age	0.237***	0.229***	0.142*
Education	0.145*	0.147*	0.090
Gender^	-0.101*	-0.096	-0.140**
COVID-19 worries		0.029	0.079
Extraversion			-0.010
Agreeableness			0.145**
Conscientiousness			0.019
Emotional Stability			0.211***
Openness			0.112*
ΔR^2^	.051***	.001	.082***
R^2^	.051***	.052***	.134***

Note. R^2^. ΔR^2^ and standardized β concern each step.

^Gender was a dichotomous variable (0 = female, 1 = male)

*p < .05

**p < .01

***p < .001

**Table 5:** Results from hierarchical regression analysis with age, gender and education (Step 1), COVID-19 worries (Step 2) and personality traits (Step 3) as predictors of expressive suppression.

	Expressive suppression		
	Model 1	Model 2	Model 3
	β	β	β
Age	0.201***	0.167**	0.198***
Education	0.002	0.015	-0.017
Gender^	0.136**	0.162***	0.122**
COVID-19 worries		0.134*	0.129**
Extraversion			-0.393***
Agreeableness			-0.050
Conscientiousness			-0.032
Emotional Stability			0.113*
Openness			-0.022
ΔR^2^	.057***	.015*	.176***
R^2^	.057***	.073***	.248***

Note. R^2^. ΔR^2^ and standardized β concern each step.

^Gender was a dichotomous variable (0 = female, 1 = male)

*p < .05

**p < .01

***p < .001

**Table 6:** Results from hierarchical regression analysis with age, gender and education (Step 1), COVID-19 worries (Step 2) and personality traits (Step 3) as predictors of optimism.

	Optimism		
	Model 1	Model 2	Model 3
	β	β	β
Age	0.273***	0.308***	0.164**
Education	0.077	0.063	0.020
Gender^	0.024	-0.002	-0.053
COVID-19 worries		-0.142**	-0.072
Extraversion			0.213***
Agreeableness			0.158***
Conscientiousness			0.075
Emotional Stability			0.301***
Openness			0.016
ΔR^2^	.058***	.017**	.183***
R^2^	.058***	.075***	.258***

Note. R^2^. ΔR^2^ and standardized β concern each step.

^Gender was a dichotomous variable (0 = female, 1 = male)

*p < .05

**p < .01

***p < .001

**Table 7:** Results from hierarchical regression analysis with age, gender and education (Step 1), COVID-19 worries (Step 2) and personality traits (Step 3) as predictors of hope—agency.

	Hope—Agency		
	Model 1	Model 2	Model 3
	β	β	β
Age	0.328***	0.339***	0.204***
Education	0.196***	0.191***	0.185***
Gender^	0.048	0.040	0.041
COVID-19 worries		-0.044	-0.024
Extraversion			0.027
Agreeableness			-0.025
Conscientiousness			0.330***
Emotional Stability			0.097*
Openness			0.022
ΔR^2^	.078***	.002	.106***
R^2^	.078***	.080***	.186***

Note. R^2^. ΔR^2^ and standardized β concern each step.

^Gender was a dichotomous variable (0 = female, 1 = male)

*p < .05

**p < .01

***p < .001

**Table 8:** Results from hierarchical regression analysis with age, gender and education (Step 1), COVID-19 worries (Step 2) and personality traits (Step 3) as predictors of hope—pathways.

	Hope—Pathways		
	Model 1	Model 2	Model 3
	β	β	β
Age	0.070	0.086	-0.035
Education	0.041	0.035	0.010
Gender^	0.087	0.075	0.050
COVID-19 worries		-0.064	-0.014
Extraversion			0.221***
Agreeableness			0.009
Conscientiousness			0.159**
Emotional Stability			0.207***
Openness			0.129**
ΔR^2^	.011	.004	.144***
R^2^	.011	.014	.159***

Note. R^2^. ΔR^2^ and standardized β concern each step.

^Gender was a dichotomous variable (0 = female, 1 = male)

*p < .05

**p < .01

***p < .001

As for emotion regulation strategies, all the predictors explained the 13.4% of the variance in CR and the 24.8% of the variance in ES and the final models were significant, *F*_(9,379)_ = 6.542, *p* < .001 and *F*_(9,379)_ = 13.911, *p* < .001, respectively.

For CR, sociodemographic factors accounted for the 5.1% of the variance, with older age, higher education and being female predicting higher CR scores. COVID-19 worries did not contribute to explaining the variance in CR scores. Personality accounted for another 8.2% of the variance. In the final model, high agreeableness, emotional stability, and openness, along with older age and being female, predicted greater CR (see Table 4).

As for ES, sociodemographic factors accounted for 5.7% of the variance, with older age and being male predicting higher ES scores. COVID-19 worries contributed to explaining a modest portion (1.5%) of the variance in ES scores, whereas personality contributed to explaining the largest portion of variance (17.6%). High emotional stability and low extraversion, along with older age, being male and higher COVID-19 worries, emerged as predictors of greater ES in the final model (see Table 5).

Concerning optimism, all predictors explained the 25.8% of the variance in the LOT-R scale, and the final model was significant, *F*_(9,379)_ = 14.676, *p* < .001. Sociodemographic factors accounted for the 5.8% of the variance, with only older age predicting higher LOT-R scores. COVID-19 worries contributed to explaining the 1.7% of the variance, with higher COVID-19 worries predicting lower LOT-R scores. In addition, personality accounted for the largest portion of variance (18.3%) on this scale, with high extraversion, agreeableness, emotional stability, and older age as predictors of greater optimism in the final model (see Table 6).

As for hope, all predictors explained the 18.6% of the variance on the hope-agency scale and 15.9% of the variance on the hope-pathways scale, and the final models were significant, *F*_(9,379)_ = 9.637, *p* < .001 and *F*_(9,379)_ = 7.942, *p* < .001, respectively.

For the hope-agency scale, sociodemographic factors accounted for the 7.8% of the variance, with older age and higher education predicting higher agency scores. COVID-19 worries did not contribute to explaining the variance on this scale, whereas personality accounted for an additional 10.6% of the variance on this scale. Conscientiousness and emotional stability, along with older age and higher education, predicted greater agency scores in the final model (see Table 7).

For the hope-pathways scale, sociodemographic factors and COVID-19 worries did not explain significant portions of variance on this scale. Instead, personality accounted for a significant portion of variance (14.4%), with high extraversion, conscientiousness, emotional stability, and openness as predictors of greater pathways scores in the final model (see Table 8).

## Discussion

The present study newly investigated the age-related differences between younger and older adults’ emotion regulation, as well as optimism and hope dispositions, 1 year after the beginning of the COVID-19 pandemic. Also, the contribution of personality traits in accounting for the emotional and psychological outcomes under such a prolonged stressful situation was examined for the first time.

Concerning emotion regulation, the results, in line with our expectations and previous findings [10], showed that older adults, compared to younger adults, reported greater reliance on CR. However, contrary to previous findings [10], older adults also reported greater reliance on ES, compared to that of their younger counterparts. It is worth stressing that outside the context of the COVID-19 pandemic, ES was found to be related to an increase in adverse affective outcomes (e.g., psychological distress) in younger adults but not in older ones [8]. In the face of situations in which stressors are not controllable, such as the prolonged COVID-19 crisis, an emotion-focused form of coping such as ES can be useful to regulate expression and experience of emotions [8, 42, 43]. Older adults are more likely able to regulate their emotions than young adults are also by matching strategies more closely to the demands of the particular context [5], thus ES might also have been, to some extent, adaptive for the former to remain as emotionally satisfied as possible in the context of the advanced COVID-19 crisis [44]. Though this is an interpretation, it is corroborated by the fact that older adults experienced COVID-19 worries (related to the ongoing third wave of contagion, as compared to previous ones) to a greater extent than younger adults did, as well as, broadly, by the albeit modest positive correlation between ES strategy and the emotional stability trait, which is related to a better ability to control negative emotional states and impulsive behaviors. Therefore, our pattern of results aligns with SST [2, 4] and further suggests that older adults have an advantage in flexibly using a combination of emotion regulation strategies to face an unusual, prolonged stressful situation such as the COVID-19 pandemic.

In line with our expectations and previous evidence acquired under the initial phases of the COVID-19 pandemic [19, 20], older adults, compared to younger ones, also displayed greater optimism. Moreover, they showed higher hope, in terms of perceived determination to reach one’s goals successfully (agency subscale), than young adults did. However, no age differences emerged for hope in terms of generating successful plans to meet goals (i.e., on the pathways subscale). Such a pattern of findings could be explained by again considering the SST framework [4]: with aging comes a shift in life perspective, priorities, and goals due to uncertain future horizons, leading older adults to be likelier than younger generations to prioritize the present over the future. Such a focus on the present facilitates older adults’ positive emotional meaning and experience, which they use to fulfill their emotional goals and preserve their well-being. If the prolonged uncertainty and the difficulty of planning for the future characteristic of the pandemic situation is manageable for older adults (more present-oriented), it might have pushed the “futures” of younger adults (who are usually more future-oriented) into uncertainty, thereby attenuating age differences -in favor of older adults and affecting younger generations more- in terms of generating successful plans and identifying ways to achieve goals. Again, these are only speculations; in this study, we did not examine individual and age-related differences in the ability to switch effectively between temporal horizons in response to situational and environmental demands, which has proven to strengthen coping skills in unexpected crises, such as the COVID-19 pandemic, and to reinforce positive expectations of the future [3].

Looking at the role of personality, in line with our expectations, personality traits, and more consistently emotional stability, influenced the emotional and psychological functioning as conceived here, above and beyond the general modest contribution of sociodemographic factors and COVID-19 worries. The results from correlation and regression analyses revealed a nuanced pattern of associations depending on the personality traits and outcome measure considered.

As for emotion regulation, in line with previous findings under the first wave of the pandemic [29], high emotional stability, but also agreeableness and openness, were associated with greater reliance on CR strategy. A greater reliance on ES strategy was associated with high emotional stability as well as low extraversion. Gubler et al. [29] did not find an association between emotional stability and ES, but such a different pattern of results could lie in the different measures adopted and the timepoint considered, the assessment of all personality traits in this study (and not only emotional stability and extraversion), as well as the sample characteristics (younger and older adults in this study and middle-aged adults in the Gubler’s et al. [29] study). Our results further suggest that the greater an individual’s ability to control negative emotional states and impulsive behaviors is (characteristics of emotional stability), the likelier that individual will be to engage various emotion regulation strategies to cope with the emotional fallout of the prolonged stressful situation at hand. Then, being open-minded and sensitive toward others and their needs (higher openness and agreeableness) seems more likely to lead individuals to rely on their cognitive resources to manage and reinterpret the emotional effect of the COVID-19 crisis using greater CR, on the one hand. It is to note, however, that the part of the variance explained by personality in CR is modest. On the other hand, being introverted and discreet (lower in extraversion) might predispose individuals to face the emotional experiences of the prolonged COVID-19 pandemic by hiding, inhibiting, or reducing ongoing emotion-expressive behaviors by adopting greater ES. However, the interplay between emotion regulation strategies, personality, and the psychological health fallout of prolonged situations with limited opportunities to engage in social activities deserves to be studied further because similar stressful situations will occur.

Concerning optimism, high emotional stability, extraversion, and agreeableness were associated with greater LOT-R scores (and they overshadowed the role of COVID-19 worries when they were added as predictors in the model). The ability to manage emotions and the outgoing nature characteristic of emotional stability and extraversion, along with the kindness and cooperativeness expressed as agreeableness, seems to predispose individuals towards embracing a more optimistic view of their future in the face of a prolonged stressful public crisis such as the COVID-19 pandemic and its related unpredictable restrictions [27].

Finally, high emotional stability and conscientiousness were associated with greater hope-agency scores, whereas high levels of all personality traits, except for agreeableness, predicted greater hope-pathways scores. These results suggest that the orderliness, perseverance, and ability to manage emotions associated with conscientiousness and emotional stability are more likely to support individuals’ determination to pursue their goals successfully and that open-minded worldviews associated with extraversion and openness also push those individuals to find ways to reach their goals beyond the constraints posed by the COVID-19 crisis.

Our analyses also showed a modest effect of education, gender and COVID-19 worries on influencing some of the outcomes considered. For instance, the effect of education on CR and hope-agency might reflect the involvement of cognitive resources in the emotion regulation strategy [33, 34] and in hope’s agency component [17]. The effect of gender on the two emotion regulation strategy scales is in line with previous evidence showing females to more likely rely on CR and males to engage more ES [7, 35], even within the COVID-19 pandemic context [36]. The effect of COVID-19 worries on ES and optimism suggests that such outcomes are likely affected by negative feelings (e.g., worry or fear) possibly arising from the prolonged crises individuals face [1, 3].

Despite these interesting findings, we should acknowledge some limitations. The cross-sectional design and the lack of a baseline timepoint throughout the emergency used in this study limited causal inferences. Moreover, a larger sample with a broader age range that also includes middle-aged participants would have helped to clarify the prolonged emotional and psychological fallout of the COVID-19 pandemic. Other factors not examined here, related to both the pandemic (e.g., contracting COVID-19 or having relatives infected by COVID-19 or experiencing negative consequences of contagion, adherence to restrictions and rules, vaccination status, or trust in the government’s management of the crisis) and to the individual (e.g., income and working status, living conditions, and lifestyle habits) and that could affect the emotional and psychological outcomes considered here, would have contributed to our study’s completeness. Our study could provide a picture of young and older adults’ psychological and emotional experiences of the COVID-19 pandemic 1 year after the start of the emergency, an aspect that has rarely been examined. It can also give insights into how older adults would differently rely on emotional and psychological resources to manage and overcome potential large-scale, extreme emergencies.

In conclusion, the results of this study further highlight that even in the later stages of such a prolonged situation, older adults, in line with the SST [4], displayed a better emotional and psychological experience of the COVID-19 crisis compared to their younger counterparts. In fact, older adults—despite experiencing COVID-19 worries to a greater extent than younger adults did—reported a more flexible and complex use of emotion regulation strategies than younger adults, as well as greater optimistic and overall hopeful attitudes. Of more interest, our findings highlight the potential of personality traits, particularly emotional stability, to have a protective effect on young and older adults’ emotional and especially psychological functioning (as reflected in optimism and hope) through prolonged phases of the COVID-19 pandemic.

Such a pattern of findings points to the need to continue monitoring emotional and psychological functioning under prolonged stressful situations such as the COVID-19 pandemic. Our findings also underscore the importance of considering personality (among other individual characteristics) to identify which individual profiles are more likely prone to experience the emotional and psychological fallout of large-scale, prolonged, extreme events. From an applied perspective, this would have practical implications for professionals who seek to implement personalized strategies and interventions to help individuals successfully use their emotional and psychological resources to cope with extraordinary crises, and more broadly for ensuring health services and opportunities that enhance individuals and community resilience in face of new large-scale emergencies.

## Supporting information

S1 Checklist STROBE statement—checklist of items that should be included in reports of observational studies. (PDF)
